# Antiglycative Activity and RAGE Expression in Rett Syndrome

**DOI:** 10.3390/cells8020161

**Published:** 2019-02-15

**Authors:** Valeria Cordone, Alessandra Pecorelli, Mascia Benedusi, Silvano Santini, Stefano Falone, Joussef Hayek, Fernanda Amicarelli, Giuseppe Valacchi

**Affiliations:** 1 Department of Life, Health and Environmental Sciences, University of L’Aquila, Via Vetoio, 67100 L’Aquila, Italy; vcordone@unite.it (V.C.); silvanojunior.santini@univaq.it (S.S.J.); stefano.falone@univaq.it (S.F.); 2 Plants for Human Health Institute, Animal Science Department, NC Research Campus, NC State University, 600 Laureate Way, Kannapolis, NC 28081, USA; apecore@ncsu.edu; 3 Department of Life Sciences and Biotechnology, University of Ferrara, Via Luigi Borsari 46, 44121 Ferrara, Italy; mascia.benedusi@unife.it; 4 Child Neuropsychiatry Unit, University General Hospital, Azienda Ospedaliera Universitaria Senese, Viale M. Bracci 16, 53100 Siena, Italy; hayekjoussef@gmail.com

**Keywords:** methylglyoxal, glyoxalases, advanced glycation end products, MeCP2, dicarbonyl stress, fibroblasts

## Abstract

Rett syndrome (RTT) is a human neurodevelopmental disorder, whose pathogenesis has been linked to both oxidative stress and subclinical inflammatory status (OxInflammation). Methylglyoxal (MG), a glycolytic by-product with cytotoxic and pro-oxidant power, is the major precursor in vivo of advanced glycation end products (AGEs), which are known to exert their detrimental effect via receptor- (e.g., RAGE) or non-receptor-mediated mechanisms in several neurological diseases. On this basis, we aimed to compare fibroblasts from healthy subjects (CTR) with fibroblasts from RTT patients (N = 6 per group), by evaluating gene/protein expression patterns, and enzymatic activities of glyoxalases (GLOs), along with the levels of MG-dependent damage in both basal and MG-challenged conditions. Our results revealed that RTT is linked to an alteration of the GLOs system (specifically, increased GLO2 activity), that ensures unchanged MG-dependent damage levels. However, RTT cells underwent more pronounced cell death upon exogenous MG-treatment, as compared to CTR, and displayed lower RAGE levels than CTR, with no alterations following MG-treatment, thus suggesting that an adaptive response to dicarbonyl stress may occur. In conclusion, besides OxInflammation, RTT is associated with reshaping of the major defense systems against dicarbonyl stress, along with an altered cellular stress response towards pro-glycating insults.

## 1. Introduction

Rett syndrome (RTT) is a neurodevelopmental disorder mostly affecting the female gender with a prevalence rate ranging from 0.43 to 1 per 10,000 live births [1,2,3]. The disease is characterized by a 6–18-month apparently normal development, followed by the loss of acquired spoken language, gait abnormalities, and replacement of purposeful hand skills with repetitive stereotypies [4,5]. RTT is caused in the 95% of the cases by sporadic de novo loss-of-function mutations in the X-linked methyl-CpG-binding protein 2 (*MECP2*) gene encoding for a nuclear protein involved in transcriptional regulation of a broad spectrum of genes, by modulating chromatin structure and RNA splicing [6,7]. Despite almost 20 years of research into the functions of MeCP2, the biochemical and molecular mechanisms leading from protein deficiency to the onset of the disorder are still not fully understood.

Nevertheless, in the last decade, several studies have highlighted a correlation between oxidative stress (OS) and a subclinical inflammatory status in RTT [8,9], showing both cellular and systemic OxInflammatory imbalances in those patients [10,11,12]. 

Oxidative stress and inflammatory responses are often associated with another type of cellular stress, the so-called dicarbonyl stress [13,14]. Dicarbonyl stress is defined as the abnormal accumulation of α-oxoaldehyde metabolites (e.g., methylglyoxal, MG), which induce glycative modifications of the main cellular macromolecules, producing advanced glycation end products (AGEs; e.g., Arg-pyrimidine and MG-derived hydroimidazolone, MG-H1), causing their dysfunction. Moreover, AGEs are able to produce adverse effects through non-receptor- and receptor-mediated (via the interaction with RAGE, the receptor for advanced glycation end products) mechanisms, leading to the activation of cellular signaling, the induction of gene expression, the production of cytokines and reactive oxygen species (ROS), as well as cell and tissue dysfunctions in ageing and pathologic conditions [15,16]. MG is a glycolytic by-product, with high cytotoxic, pro-oxidant and pro-apoptotic power [17,18,19]. MG accumulation can promote ROS generation (e.g., the conversion of aminoacetone to MG yields hydrogen peroxide) and, at the same time, free radical-related reactions can take part in its formation (e.g., ROS-related glucose autoxidation and lipid peroxidation lead to MG production) [18,20,21]. Methylglyoxal exerts GSH-depleting effect by affecting several glutathione-related enzymes (e.g., glutathione reductase, glutathione peroxidase, glutathione synthetase, and γ-glutamylcysteine synthetase), and inhibiting the activity of other antioxidants enzymes [17,22,23]. MG can also induce mitochondrial permeability transition and decrease the complex III activity, thus impairing mitochondrial functions [23,24,25]. 

Intracellular MG is mainly metabolized by the glyoxalase detoxifying system, consisting of the glutathione-dependent enzyme glyoxalase 1 (GLO1), which converts MG in S-ᴅ-lactoylglutathione, and glyoxalase 2 (GLO2), leading to ᴅ-lactate and then reconstitution of the GSH pool [26].

Several studies have reported the role of dicarbonyl stress and AGEs in neurodegenerative pathologies [27,28] and in neurodevelopmental disorders [29]. The glycation of abnormal accumulating proteins (i.e., amyloid beta, tau, prions, and transthyretin) in brains of patients with neurodegenerative diseases is supposed to be associated with crosslinking formation, leading to more stable protein aggregates and then exacerbating their neurotoxicity [30,31]; accordingly, the positive modulation of AGEs-targeting detoxifying systems, such as GLO1, has been demonstrated to contrast cognitive decline in a mouse model of Alzheimer’s disease (AD) [32]. The dicarbonyl glycation of cellular proteins is involved in the modulation of inflammatory protein expression (through the activation of RAGE) [33,34], being therefore a key component in the neuroinflammation processes of several neurological diseases, including AD, Parkinson’s disease, and amyotrophic lateral sclerosis [35,36,37,38]. Indeed, AGEs production and/or RAGE activation are now considered to be a promising drug target for patients who are affected by these disorders [28]. 

To the best of our knowledge, the RTT, which is characterized by a generalized state of oxidative stress and subclinical inflammation [8,11,39,40] has never been linked to dicarbonyl stress. The aim of our study was to investigate whether RTT could be associated to an imbalances in glycative homeostasis. The experimental model used in this work consisted of primary fibroblasts isolated from RTT patients, a model that has already been shown to be reliable for studying the molecular mechanisms involved in neurological disorders, including RTT [39,41,42].

## 2. Materials and Methods

### 2.1. Antibodies

Abcam (Cambridge, UK) provided the anti-glyoxalase 1 (GLO1) (cat. Ab171121; dilution: 1:1,000) and anti-glyoxalase 2 (GLO2) (cat. Ab154108; dil. 1:250) antibodies. Santa Cruz Biotechnology, Inc. (Santa Cruz, CA, USA) provided the anti-RAGE (cat. sc-365154; dilution: 1:250) antibody. The anti-α-tubulin antibody (cat. T5168; dilution 1:8000) and the peroxidase-conjugated anti-mouse secondary antibody (cat. A9044; dil. 1:10,000) were purchased from Sigma–Aldrich (Milano, Italy). Vector Laboratories (Peterborough, UK) provided the peroxidase-conjugated anti-rabbit secondary antibody (cat. PI1000; dil. 1:1000).

### 2.2. Study Approval

The study samples consisted of female patients with classical RTT (N = 6; age: 20 ± 3.8, expressed as mean ± SD) and healthy female age-matched controls (N = 6). All the patients were constitutively admitted to the Child Neuropsychiatry Unit of the University Hospital of Siena (Siena, Italy). The diagnosis of RTT and selection criteria (inclusion/exclusion) were set in accordance with revised RTT nomenclature consensus [4]. The study was designed and performed according to the Code of Ethics of the World Medical Association (Declaration of Helsinki), and the protocol was approved by the Ethics Committee of Institutional Review Board of University Hospital, Azienda Ospedaliera Universitaria Senese (AOUS), Siena, Italy. Informed consents were obtained in written form from either the parents or the legal tutors of the participants.

### 2.3. Blood Sampling

Blood was collected in heparinized tubes, and all manipulations were carried out within 2 h after sample collection. The blood samples were centrifuged 2400× *g* for 15 min at 4 °C, and plasma was collected. Patients’ characteristics are summarized in Table 1. The severity score was assessed following the CSS (Clinical Severity Score) by Dr. Joussef Hayek.

### 2.4. Human Fibroblasts Culture

Control skin biopsies were obtained during routine health checks or by donations, while skin biopsies from RTT patients were carried out during the clinical check-ups. The isolation of human skin fibroblasts was performed by a 3-mm skin punch biopsy, as previously described [43]. Before the experimental procedure, fibroblasts were identified with positive staining for Vimentin and tested for mycoplasma contamination. Cells were cultured with 10% (*v*/*v*) fetal bovine serum-supplemented Dulbecco′s Modified Eagle′s Medium (DMEM) (cat. 35-011-CV and cat. 10-014-CV, respectively, all from Corning, New York, NY, USA), containing antibiotics (100 IU/mL penicillin, 100 mg/mL streptomycin) (cat. 30-002-CI, from Corning, New York, NY, USA) and incubated in humidified atmosphere (5% CO_2_) at 37 °C. In all experiments, fibroblasts were used between the third and fifth passage in vitro.

### 2.5. Cell Extract Preparation for Enzymatic Activity Assessments

The lysis of sub-confluent cells (3 × 10^7^ cells/mL) was performed in 100 mM KH_2_PO_4_, 1.5 mM dithiotreitol (DTT) and 1 mM Ethylenediaminetetraacetic acid (EDTA) (pH 7) extraction buffer for both glyoxalase 1 and glyoxalase 2 enzymatic activity assays. Cell suspensions were homogenized and centrifuged at 16,000× *g* for 30 min at 4 °C. Protein extracts were used for enzymatic activity and for the quantification of total protein concentration, by using the Bradford assay (cat. 500-0006, Bio–Rad Laboratories, Hercules, CA, USA) and bovine serum albumin (BSA) as the standard [44]. All spectrophotometric readings were carried out in triplicate by using a Lamba25 UV-VIS spectrophotometer (PerkinElmer, Inc., Waltham, MA, USA).

### 2.6. Glyoxalase 1 (GLO1) Activity

The GLO1 (EC 4.4.1.5) activity was measured at 240 nm at 25 °C, by recording the appearance of (R)-*S*-lactoylglutathione, as described by Mannervik et al. [45]. The reaction mixture consisted of 1 mM GSH (cat. G4251, Sigma–Aldrich) and 2 mM methylglyoxal (cat. M0252, Sigma-Aldrich). One unit of GLO1 activity was defined as 1 μmol of (R)-*S*-lactoylglutathione formed/min. Readings were performed in quadruplicate.

### 2.7. Glyoxalase 2 (GLO2) Activity

GLO2 (EC 3.1.2.6) activity was evaluated at 240 nm at 25 °C, by recording the disappearance of 0.3 mM (R)-*S*-lactoylglutathione (cat. L7140, Sigma–Aldrich), as described by Guha et al. [46]. One unit of GLO2 activity was defined as 1 μmol of lactoylglutathione hydrolyzed/min. Readings were performed in quadruplicate.

### 2.8. Western Immunoblot Analysis

Sub-confluent fibroblasts were lysed (2 × 10^7^ cells/mL) in RIPA buffer (cat. R0278, Sigma–Aldrich), supplemented with 1% (*v*/*v*) protease inhibitors (cat. P8340, Sigma–Aldrich) and 1% (*v*/*v*) phosphatase inhibitors (cat. P2850 and P5726, Sigma–Aldrich). Cell lysates were centrifuged at 16,000× *g* for 30 min at 4 °C, and supernatants were assayed for total protein concentration, by using the BCA Protein Assay Kit and BSA as the standard (cat. PR23225, EuroClone, Milan, Italy). Samples were denatured and run in triplicates on 12% polyacrylamide. Bands were then transferred onto polyvinylidene difluoride (PVDF) membranes by electrophoretic transfer (as previously described [43]). After the blocking of non-specific binding sites at room temperature for 1 h with 5% (*w*/*v*) Blotting-Grade Blocker (cat. 170-6404, Bio-Rad Laboratories), in Tris-buffer saline containing 0.05% (*v*/*v*) Tween-20 (cat. P5927, Sigma–Aldrich) (TBS-T), membranes were incubated overnight with the primary antibodies diluted in TBS-T (see Section 2.1. for the antibodies used), and then with anti-rabbit or anti-mouse peroxidase-conjugated secondary antibodies diluted in TBS-T for 2 h. The protein bands were detected by using Enhanced Chemiluminescent Substrate Kit (cat. EMP001005, EuroClone) and Alliance LD2 system (UVItec Limited, Cambridge, UK). Images of bands were analyzed by using Nonlinear Dynamics TotalLab software (TotalLab Ltd, Newcastle upon Tyne, UK). Data were normalized against α-tubulin, and results were given as arbitrary units.

### 2.9. RNA Extraction and Real-Time Reverse Transcriptase-Polymerase Chain Reaction (RT-PCR) Analysis

Total RNA was extracted from fibroblasts by using Ribospin kit (cat. 304-150, GeneAll Biotechnology CO., Ltd, Seoul, Korea), and contaminant genomic DNA was digested by Riboclear plus (cat. 313-150), following the supplier’s instructions (GeneAll Biotechnology CO., Ltd). According to the protocol of the reverse transcription kit (cat. NP100041, OriGene Technologies, Inc., Rockville, MD, USA), RNA (1 μg) was converted into complementary DNA (cDNA). The cDNA (diluted 1:10) was used for the SensiFastTM SYBR-based polymerase chain reaction (PCR) step (cat. BIO-92005, Bioline, London, UK) in an Applied Biosystems 7300 system (ThermoFisher Scientific, Inc., Rockford, IL, USA). Primers were synthetized by IDT Integrated DNA Technologies, Inc. (Coralville, IA, USA) (Table 2). Amplification steps were set as follows: initial single denaturation at 95 °C for 2 min, and 40 cycles of 95 °C for 5 s and 60 °C for 30 s. In order to verify the possible co-amplification of unspecific targets, melting curves were performed for all of the primer pairs (95 °C for 15 s, 60 °C for 1 min, 95 °C for 15 s, and 60 °C for 15 s). Gene expression was calculated by using the ΔΔCt method [47], using the transcript coding for ribosomal protein L13a (rpl13a) as the reference, and one of the controls as the internal calibrator. Each sample was processed by analyzing three replicates.

### 2.10. Detection of MG–Protein Adducts by ELISA Assay

MG-H1 protein adducts were measured by using a competitive enzyme-linked immunosorbent assay (ELISA) kit (Cell BioLabs, Inc., cat. STA-811, San Diego, CA, USA). Briefly, a MG-conjugate was coated on the ELISA plate, as recommended by the manufacturer. Control (CTR) and RTT samples or MG–BSA standards were added in triplicate to the pre-adsorbed plate. An anti-MG specific monoclonal antibody was incubated for 1 h at room temperature, followed by washes and an incubation with horseradish peroxidase (HRP)-conjugated secondary antibody, as specified by the kit’s manufacturer. The contents of MG-H1 adducts in the protein samples were determined through a 4P-logistic regression equation by comparing the absorbance at 450 nm with that of the MG–BSA standard curve. A microplate reader, Victor3 (PerkinElmer, Inc., Waltham, MA, USA), was used for readings. Results were given as μg MG-H1/mg of total proteins.

### 2.11. Exogenous MG-Related Cytotoxicity

CTR and RTT fibroblasts were seeded (5000 cells/cm^2^) in quadruplicate in 10% FBS-supplemented DMEM medium, containing 100 IU/mL penicillin and 100 μg/mL streptomycin. After 48 h, cells were starved with 1% FBS-containing medium for 15 h to minimize cell proliferation, and then incubated with or without 650 μM methylglyoxal, dissolved in 0.1% FBS-supplemented medium, to avoid MG-protein binding. After 24 h, cells were detached with Trypsin/EDTA (cat. 25-052-CI, Corning). Viable cells were counted in a hemocytometer chamber by Trypan blue 0.4% (*w*/*v*) staining (cat. 15250-061, Life Technologies Italia, Monza, Italy). The MG concentration (650 μM) was chosen according to a dose-response curve (0, 0.25, 0.5, 0.65, 0.8, 1, and 2 mM), that was carried out to find the concentration able to reduce viable cells by 30% (IC_30_). Results were given as viable and dead cells (%).

### 2.12. Statistics

Statistical analyses were performed by using Statsoft Statistica10 and GraphPad Prism 6 software. *t*-test for independent groups, or Two-way Analysis of variance (ANOVA) and post hoc Tukey’s test were used. The null hypothesis was rejected with a *p* value of less than 0.05. All data were expressed as means ± standard deviations (SD).

## 3. Results

### 3.1. Evaluation of Glyoxalase (GLO1 and GLO2) Expression and Activity in RTT Cells

**Figure 1:**
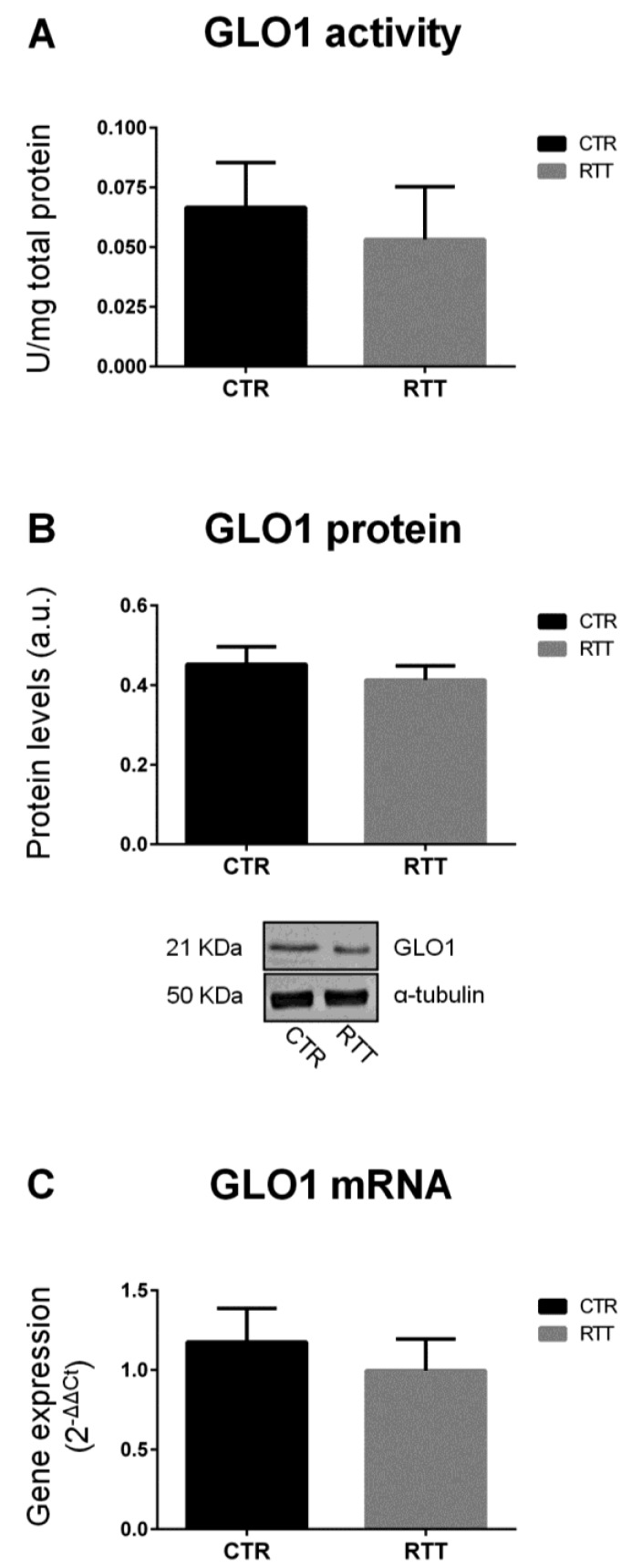
Evaluation of glyoxalase 1 pattern. (**A**) GLO1 specific activity; (**B**) GLO1 protein levels, with representative (inverted) Western blots; (**C**) glo1 gene expression levels. Data of real time RT-PCR were given as 2^−^^ΔΔCt^, using rpl13a as the reference, and one of the controls as the internal calibrator. All the data were expressed as means ± SD. CTR, control; RTT, Rett syndrome. Data were analyzed by a *t*-test for independent groups.

The accumulation of methylglyoxal is prevented by the glyoxalase system, which involves two enzymes, GLO1 and GLO2. As shown in Figure 1, fibroblasts from RTT patients exhibited unchanged levels of GLO1 specific activity, and protein and gene expression (Figure 1A–C, respectively), as compared to CTR.

**Figure 2:**
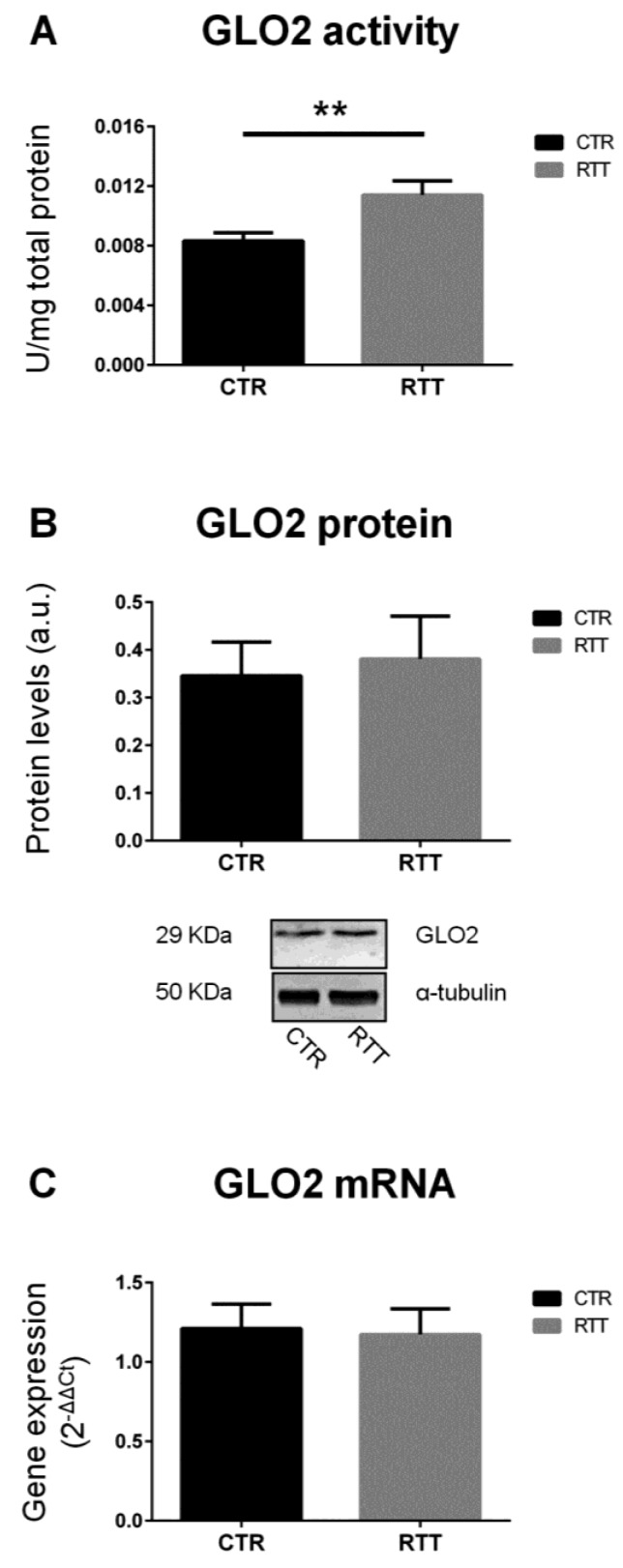
Assessment of glyoxalase 2 pattern. (**A**) GLO2 specific activity; (**B**) GLO2 protein levels, with representative (inverted) Western blots; (**C**) *glo2* gene expression levels. Data of real time RT-PCR were given as 2^−^^ΔΔCt^, using rpl13a as the reference, and one of the controls as the internal calibrator. All the data were expressed as means ± SD. CTR, control; RTT, Rett syndrome. ** *p* < 0.01. Data were analyzed by a *t*-test for independent groups.

On the other hand, as shown in Figure 2A, RTT fibroblasts revealed a statistically significant increase of the specific activity of GLO2 (*p* < 0.01), the rate-limiting enzyme in the GLOs system [48,50]. No statistically differences were observed in GLO2 protein and mRNA levels (Figure 2B,C, respectively).

### 3.2. Effect of MG on Cellular Viability

**Figure 3:**
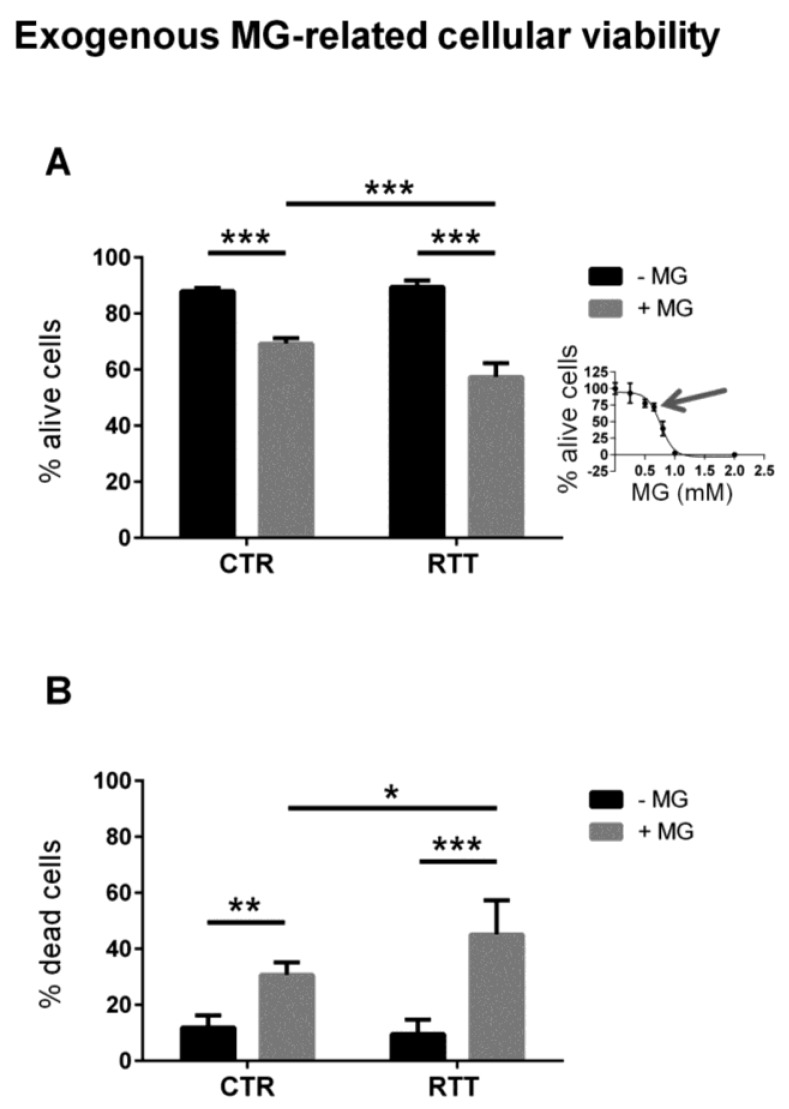
Cell survival from a 24-h exogenous MG challenge. (**A**) Cell viability of CTR and RTT fibroblasts, upon MG treatment; (**B**) cell death of CTR and RTT fibroblasts following MG challenge. Values were expressed as means ± SD. The chosen MG concentration (650 μM) represented the 30% reduction of live cells (IC_30_, indicated by the arrow), calculated through a 4P-logistic regression curve derived from a dose-response curve obtained by incubating cells with MG concentrations ranging from 0 to 2 mM (inset diagram). CTR, control; RTT, Rett syndrome; MG, methylglyoxal. * *p* < 0.05; ** *p* < 0.01; *** *p* < 0.001. Results were analyzed by Two-way ANOVA, with post hoc Tukey’s multiple comparisons test.

In order to evaluate the cellular susceptibility to methylglyoxal toxicity, fibroblasts were treated with exogenous MG. As reported in Figure 3A, cells from both control and RTT patients showed decreased cell viability upon MG treatment (*p* < 0.001). However, RTT fibroblasts were significantly more susceptible to MG than control cells (57.3% vs. 69.3% of live cells, respectively). As expected, the percentage of dead cells was significantly higher in MG-challenged RTT fibroblasts, than in MG-treated control cells (Figure 3B).

### 3.3. MG-Dependent Protein Damage in RTT Cells

**Figure 4:**
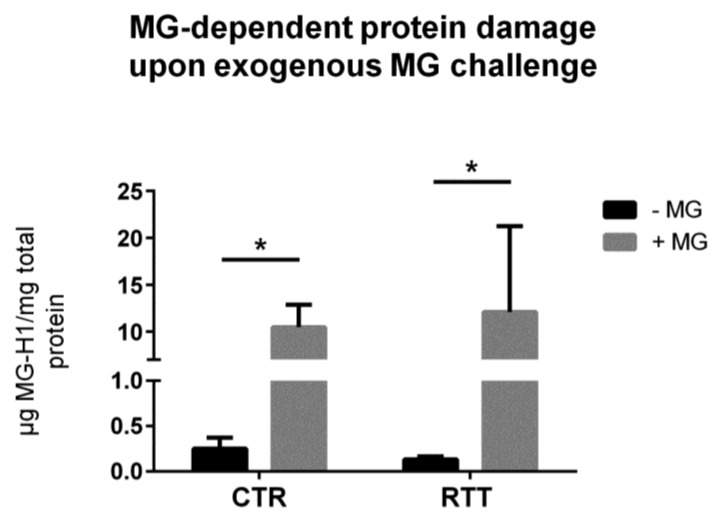
Assessment of methylglyoxal-derived hydroimidazolone 1 (MG-H1) levels, following a 24 h exogenous MG challenge. Data were given as means ± SD. CTR, control; RTT, Rett syndrome; MG, methylglyoxal. * *p* < 0.05. Results were analyzed by two-way ANOVA, with post hoc Tukey’s multiple comparisons test.

Methylglyoxal is able to react with arginine and lysine side chains, and to modify proteins irreversibly [51]. In the present work, the irreversible methylglyoxal–protein adduct MG-H1 [51,52] was analyzed. RTT fibroblasts showed statistically unchanged intracellular levels of MG-H1, as compared to untreated controls (Figure 4). As expected, the MG treatment caused a statistically significant increase of MG-H1 levels in both CTR and RTT cells (*p* < 0.05). 

### 3.4. Evaluation of Cellular RAGE Levels

**Figure 5:**
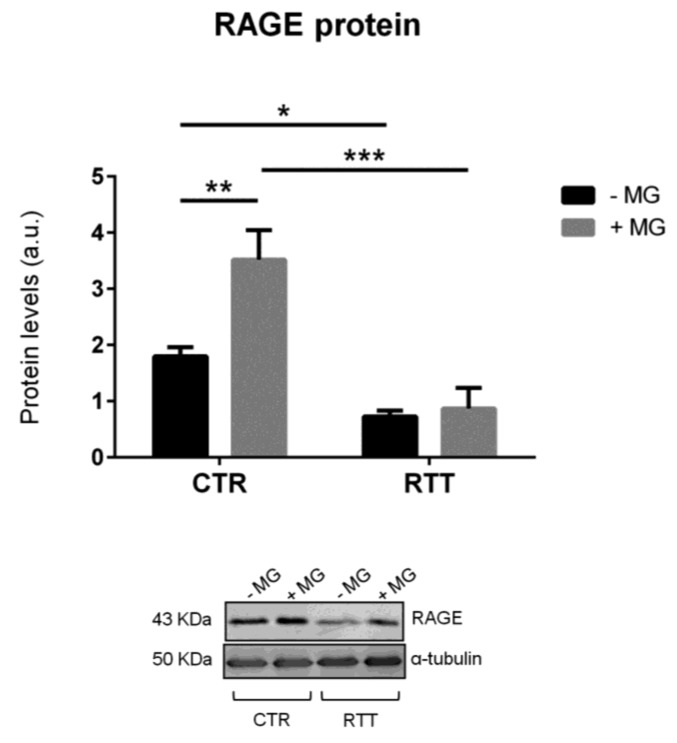
Evaluation of cellular RAGE protein levels in MG-challenged fibroblasts. Data were expressed as means ± SD. Representative (inverted) Western blots were reported. CTR, control; RTT, Rett syndrome; MG, methylglyoxal. * *p* < 0.05; ** *p* < 0.01; *** *p* < 0.001. Results were analyzed by two-way ANOVA, with post hoc Tukey’s multiple comparisons test.

As MG-H1 is a ligand of the receptor for advanced glycation end products (RAGE) [53], we analyzed RAGE protein expression. RAGE baseline levels in RTT cells were significantly lower (*p* < 0.05), with respect to CTR fibroblasts, whereas, MG-challenge was able to increase RAGE levels only in CTR fibroblasts (*p* < 0.01), with no variation detected in RTT cells (Figure 5).

### 3.5. MG-Dependent Dicarbonyl Damage in Plasma 

**Figure 6:**
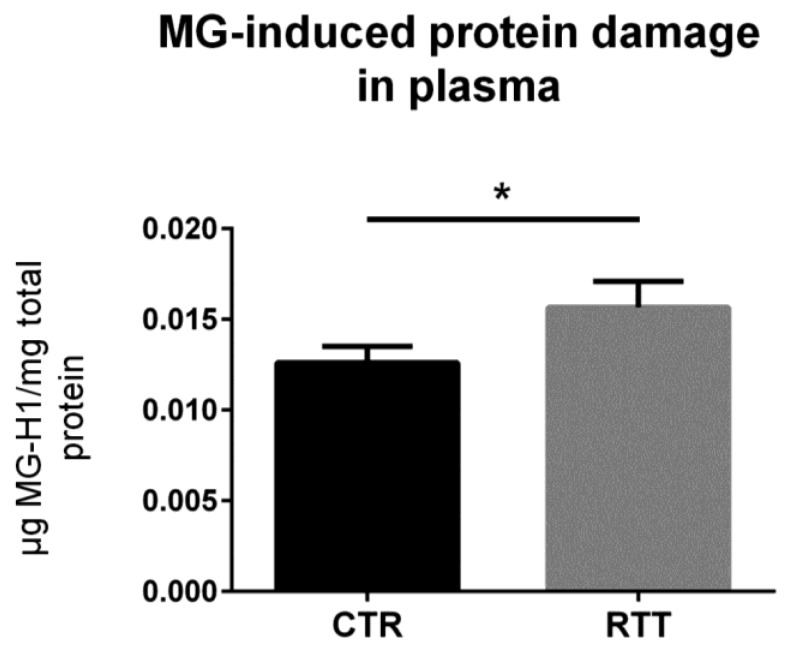
Assessment of dicarbonyl damage by the analysis of MG-H1 levels in plasma from RTT patients. Data were given as means ± SD. CTR, control; RTT, Rett syndrome. * *p* < 0.05. Data were analyzed by a *t*-test for independent groups.

To confirm the clinical relevance, the circulating levels of MG-dependent protein damage were analyzed in plasma samples from RTT patients. As revealed by the competitive immunoassay, plasma from RTT patients showed increased levels of MG-H1 (*p* < 0.05), as compared to plasma from CTR (Figure 6).

## 4. Discussion

Based on the strong link that has been shown between RTT and oxidative stress, the aim of the present work was to further investigate whether the dicarbonyl stress pathway is involved in RTT-altered redox homeostasis. In order to answer this question, we used primary skin fibroblasts isolated from RTT patients, a reliable model to study molecular mechanism involved in genetic diseases, as they perfectly mirror the disease’s background [39,41,42]. Overall, our data suggest an alteration of the GLOs system in RTT cells. Indeed, RTT fibroblasts, although not significant, showed a trend in decreased activity, protein levels, and mRNA expression of glyoxalase 1 (GLO1) with respect to CTR. In addition, there was a statistically significant increase of GLO2 specific activity in RTT fibroblasts. GLO2 catalyzes the hydrolysis of *S*-ᴅ-lactoylglutathione to form ᴅ-lactic acid and glutathione, and represents the rate-limiting step in MG removal [50]. Therefore, an increase in its activity may ensure an improved degree of detoxification against MG overall, and this could explain the unchanged levels of MG-H1 in RTT fibroblasts. In addition, the observed increase of GLO2 specific activity may also assure a more rapid rate of GSH recycling, which may help to compensate the GSH depletion that has been shown to be present in RTT cells [39,54]. Furthermore, a recent study from Ercolani et al. suggested a possible additional role of GLO2 that, interacting with a target protein, can induce S-glutathionylation using its substrate *S*-ᴅ-lactoylglutathione [55]. *S*-Glutathionylation is a post-translational modification (PTM), that can lead to store reduced glutathione during oxidative challenges, or even to protect protein thiol groups from irreversible oxidation [55,56]. Interestingly, since no significant changes in GLO2 protein and transcript levels were found in RTT cells, the increase of GLO2 enzymatic activity suggests that a possible PTM of GLO2 protein may occur in RTT, leading to a more efficient enzymatic activity. For instance, the susceptibility of the GLO system effects by PTM, especially in an altered redox homeostasis status, has been already reported [57]. Thus, future investigations are needed to better clarify the nature of the eventual PTM involved in GLO2 up-regulation observed in RTT. 

Pathologies with oxidative stress conditions may present a low efficiency of both mitochondrial metabolism and respiratory chain activity, and this may cause a switch towards the glycolysis, to improve energy production [58]. In RTT fibroblasts, glycolysis has been found to be impaired [39], thus leading to the hypothesis of a possible accumulation of triose phosphates (e.g., glyceraldehyde 3-phosphate and dihydroxyacetone phosphate), which are known to be potential precursors of MG [26]. In coherence with this notion, α-enolase, whose catalysis converts 2-phosphoglycerate to phosphoenolpyruvate, resulted in the alteration of RTT, thus confirming that glycolysis was compromised [59]. MG can also derive from non-glycolytic pathways, including lipid peroxidation and myeloperoxidase-catalyzed catabolism of amino acids [60]. Previous papers have shown the involvement of increased ROS production and lipid peroxidation levels in RTT cells [39,59], suggesting a possible role in contributing to MG generation [60,61].

Taken together, our results suggest that RTT fibroblasts seem to adapt to a possible glycative burden through a positive modulation of the limiting step of the MG removal process.

As is well known, in vivo MG levels may depend on both altered ratios between production/removal and dietary intake [26].

Is the system sufficiently protected from further pro-glycating challenges? Our experiments revealed that RTT cells underwent more pronounced cell death after exogenous MG stress, as compared to MG-challenged controls, thus suggesting that in RTT cells, the glyoxalase scavenging system is unable to efficiently protect fibroblasts from further activation. Surprisingly, no differences in MG-dependent protein damage were detected when we compared CTR and RTT cells exposed to MG. A possible explanation might be that in the presence of an exogenous MG-induced dicarbonyl stress, a minor sub-population of RTT cells with insufficient capacity to properly counteract the challenge did not survive; conversely, a prevalent cell sub-population with higher adaptive ability resisted the pro-glycating treatment, and presented levels of MG-induced protein damage that were comparable to those observed in MG-challenged control cells. The fact that cells within female subjects are a mosaic of two different populations due to the X-chromosome inactivation (XCI) could support this hypothesis. Fibroblasts from RTT patients are mosaic for the expression of the heterozygous *MECP2* mutation [62,63,64,65,66], and our results could likely be linked to this mechanism of gene dosage compensation. 

High concentrations of MG-H1 and AGEs in general are known to induce an over-expression of cellular RAGE, thus activating a RAGE-mediated response, which leads to the stimulation of a number of signaling cascades (including Jak/Stat, NADPH oxidase, mitogen activated protein kinase (MAPK)), such as p38, extracellular regulated (ERK)-1/2 and c-Jun N-terminal kinase (JNK)), the induction of gene expression, and the production of cytokines and ROS [15,67].

On this basis, we evaluated the expression of the receptor RAGE. As expected, CTR fibroblasts showed a marked increase of RAGE levels after MG challenge. It has been shown that AGEs are able to trigger RAGE over-production in a positive feedback cycle, promoting a pro-inflammatory response [68,69,70]. Surprisingly, our results revealed a completely different situation. In fact, not only did RTT cells show a significant lower RAGE expression in basal condition, but they also did not respond to MG treatment. 

We hypothesize that, since an activation of the AGEs/RAGE pathway would lead to further pro-inflammatory effects, a form of adaptation (i.e., reduced protein basal RAGE levels) may occur in RTT fibroblasts in an attempt to avoid the enhancement of subclinical inflammation and the highly stressed conditions (i.e., oxidative stress and mitochondrial dysfunction) that characterize the disease [8,39,71,72]. In light of this, we evaluated the MG-H1 levels in plasma of RTT patients as a marker of systemic MG-dependent dicarbonyl stress, finding significantly increased levels of circulating MG–protein adducts in RTT patients. This supports our hypothesis of a possible adaptive response of RTT cells, which are subjected to both an OxInflammatory status and systemic dicarbonyl stress. 

In conclusion, our work brings new insights of the possible oxidative picture present in RTT patients. Our data support the idea that the chronic OxInflammation that is typical of RTT, together with systemic dicarbonyl stress, may contribute to inducing a deregulation of the cellular stress response mechanism (both the MG-related detoxifying system and RAGE-dependent pathway), leading cells to an adaptive homeostatic response. This adaptation yields RTT cells unable to respond to further pro-glycating/pro-inflammatory stimuli, making the cells unable to cope with further eventual noxious challenges.

## Figures and Tables

**Table 1:** Clinical characteristics of Rett syndrome (RTT) patients included in this study. AA = aminoacids; CSS = Clinical Severity Score.

Patient	Mutation Type	AA Change	Age (years)	CSS Total Score
1	Early Truncating	R168X	6	35
2	Early Truncating	K144fs	22	37
3	Deletion	c.806delG	11	15
4	Missense	R133C	30	9
5	Missense	D156E	8	26
6	Early Truncating	R270X	6	25
7	Early Truncating	R255X	29	12
8	Missense	T158M	12	14
9	Missense	T158M	24	31
10	Large Deletion		22	33
11	Early Truncating	R270X	14	22
12	Missense	R106C	9	19

**Table 2:** Primer sequences used for amplicon generation.

	Forward	Reverse	Reference
*Glo1*	5’-AAGCAGGCTAGGCATGTGAA-3’	5’-CCCAAGAGCCAAGAGCACAA-3’	[48]
*Glo2*	5’-CTGCCCTGACCGACAACTAC-3’	5’-GTTTCACCCCGTGCTTTCTC-3’	[48]
*Rpl13a*	5’-CCCGTCCGGAACGTCTATAA-3’	5’-CTAGCGAAGGCTTTGAAATTCTTC-3’	[49]
